# Does China’s corporate two-way foreign direct investment mitigate environmental pollution?

**DOI:** 10.1371/journal.pone.0333935

**Published:** 2025-10-07

**Authors:** Feng Yang, Tingwei Chen, Linlin Han, Susu Wang

**Affiliations:** 1 School of Business, Liaocheng University, Liaocheng Shandong, China; 2 School of Economics and Management, Shandong Youth University of Political Science, Jinan Shandong, China; 3 School of Economics, Shandong Normal University, Jinan, Shandong, China; 4 School of Economics and Management, Qilu Normal University, Jinan, Shandong, China; Sichuan University, CHINA

## Abstract

The global push for low-carbon growth highlights the urgent need to examine how corporate internationalization shapes environmental performance. However, the environmental implications of corporate two-way foreign direct investment (CTFDI, integrating inward and outward FDI) remain insufficiently explored. This study investigates whether CTFDI mitigates corporate pollution emissions in line with the pollution halo hypothesis. This study employs a multiple fixed-effects OLS model using 43,410 firm-year observations from 2,894 A-share listed Chinese firms over 2008–2022. The results indicate that CTFDI significantly reduces emissions, with a one-unit increase associated with an average 0.15% decline. Mechanism analysis demonstrates that research and development investment and the adoption of digital and intelligent technologies are primary channels through which CTFDI exerts this effect. Heterogeneity analysis further reveals that non-state-owned enterprises and firms in non-polluting industries experience more pronounced benefits. Overall, the findings provide robust empirical evidence supporting the pollution halo hypothesis from the perspective of two-way FDI and highlight the role of economic openness in advancing green corporate development.

## 1. Introduction

Amid escalating global climate challenges, governments worldwide are increasingly enforcing stricter environmental regulations to advance the agenda of sustainable development [1,2]. Given that countries with high carbon emissions play a pivotal role in achieving global climate goals, China’s position is particularly critical. As the world’s largest developing economy and a major carbon emitter, China bears both the responsibility and the opportunity to contribute substantially to global emission reductions. Consequently, it has actively introduced a comprehensive set of environmental policies and green finance measures to curb pollution and accelerate its green transition [3,4]. Within dual global-domestic framework, corporate investment decisions in China emerge as a critical driver of the country’s environmental performance, thereby exerting consequential impacts on global climate dynamics. Within this evolving regulatory landscape, corporate investment decisions have become pivotal in shaping environmental outcomes [5–7]. When environmental considerations are effectively embedded into investment strategies, corporations can significantly contribute to emission reductions and ecological sustainability [8]. In contrast, neglecting environmental factors in corporate planning may exacerbate ecological degradation and hinder long-term environmental goals [9].

Under globalization, foreign direct investment (FDI) and outward direct investment (ODI) have become core strategies for corporate internationalization, enabling corporations to access advanced technologies, global markets, and cost efficiencies [10]. Yet their environmental consequences remain highly contested. On the positive side, FDI can enhance environmental performance by facilitating technology transfer [11], improving energy efficiency [12], and fostering green innovation [13], supporting the “pollution halo” hypothesis [14–16]. Similarly, ODI may promote cleaner production through reverse technology spillovers and the absorption of advanced green practices from host countries [17]. Conversely, international capital may also gravitate toward regions with weaker environmental regulations, consistent with the “pollution haven” hypothesis [18–20]. FDI can exacerbate environmental degradation in either host or home countries when environmental regulations are weak or poorly enforced [18,19,21], while ODI risks increasing domestic carbon emissions by expanding production capacity and investment intensity [22].

Building on these mixed outcomes of FDI and ODI, recent scholarship has begun to focus on corporate two-way FDI behavior(CTFDI), which involves simultaneous engagement in both FDI and ODI. This emerging line of research recognizes that the interplay of inward and outward investment may create environmental effects distinct from those examined in isolation [23]. CTFDI can generate positive effects because it facilitates technology acquisition, reverse spillovers, and global environmental integration, which is consistent with the “pollution halo” hypothesis. However, it may also intensify ecological pressures by expanding production, misallocating resources, or relocating polluting activities to countries with weaker environmental regulations, reflecting the risks described in the “pollution haven” hypothesis [24–26]. These contrasting possibilities highlight the need for further empirical investigation.

Despite these contrasting theoretical possibilities, the relationship between CTFDI and environmental performance at the firm-level remains unclear. Existing evidence is mixed and largely based on national or industry-level analyses. Tian et al. (2023) [27] find that industry-level two-way FDI reduces emissions, whereas Jiang et al. (2021) [28] report that bilateral investment can increase emissions in certain regions. By conducting a firm-level analysis, this study integrates these two perspectives and provides micro-level evidence on how CTFDI mechanisms, including technology transfer, investment in green research and development (R&D), and digital technology adoption, shape corporate environmental performance. This approach helps reconcile the seemingly conflicting findings by demonstrating how corporate environmental management can encompass both long-term spillover benefits and short-term expansion pressures. Most studies are conducted at the national or industry level, which provides limited insights into the corporate mechanisms through which CTFDI affects environmental outcomes. Current literature mainly focuses on financial, strategic, and innovation outcomes, such as performance improvement and exchange rate exposure, while few studies directly examine environmental impacts, including carbon emissions and pollution mitigation [29,30]. Clarifying this firm-level relationship is therefore essential to determine whether CTFDI serves as a driver of environmental upgrading or a channel for pollution displacement.

To bridge this gap, this study aims to examine whether CTFDI facilitates pollution reduction and improves environmental performance in Chinese A-share listed firms. In examining this question, the study also investigates the mechanisms through which CTFDI affects corporate environmental outcomes. This research presents three core contributions, each carrying potential implications for academic inquiry, policy development, and practical application. First, this study advances the ongoing discourse on the pollution halo versus pollution haven hypotheses by providing firm-level evidence on the environmental impacts of CTFDI. By integrating corporate investment behavior with the coordinated dynamics of inward and outward FDI, this study advances theoretical discourse in international business and environmental economics and provides rigorous empirical evidence on the causal pathways through which two-way FDI shapes environmental performance. Second, this study elucidates how China’s dual circulation strategy can be operationalized at the corporate level through CTFDI. Recognizing that the attainment of national environmental objectives ultimately hinges on firm-level implementation, the analysis contributes to constructing a conceptual and policy bridge between two-way FDI coordination and environmental governance. This framework thereby highlights the alignment of macro-level strategic priorities with micro-level corporate actions, reinforcing the policy salience of CTFDI. Third, the findings suggest that corporations can achieve sustainable development by integrating emission reduction targets into their long-term strategic agendas, rather than treating them as isolated compliance requirements. Such an integrative approach allows environmental improvement to emerge organically as part of business development, offering practical guidance for corporations aiming to enhance energy efficiency and environmental performance in alignment with their core objectives.

This study is primarily guided by the pollution halo hypothesis, which posits that FDI can promote environmental upgrading by facilitating the transfer of advanced environmental technologies and management practices [13,31]. In the context of CTFDI, this hypothesis implies that corporations engaged in both FDI and ODI are more likely to internalize international environmental standards and adopt green strategies across borders. Furthermore, informed by the resource-based view (RBV) and institutional theory, this study contends that CTFDI serves as a channel through which corporations acquire environmentally strategic capabilities, including green innovation and digital infrastructure, and adapt their behaviors in response to regulatory and normative pressures.These conceptual foundations are operationalized through a mechanism analysis, empirically investigating whether green R&D investment and digital technology adoption function as mediating channels through which CTFDI enhances environmental outcomes.

The remainder of this study is organized as follows. Section 2 reviews the mechanism and research hypotheses. Section 3 introduces the data and methodology. Section 4 presents the empirical results, including baseline regressions, endogeneity tests, robustness checks, heterogeneity analysis, and mechanism testing. Section 5 discusses the key findings and their policy implications. Section 6 concludes the paper by summarizing the main conclusions, implications, limitations, and directions for future research.

## 2. Mechanism and hypothesis

**Fig 1:**
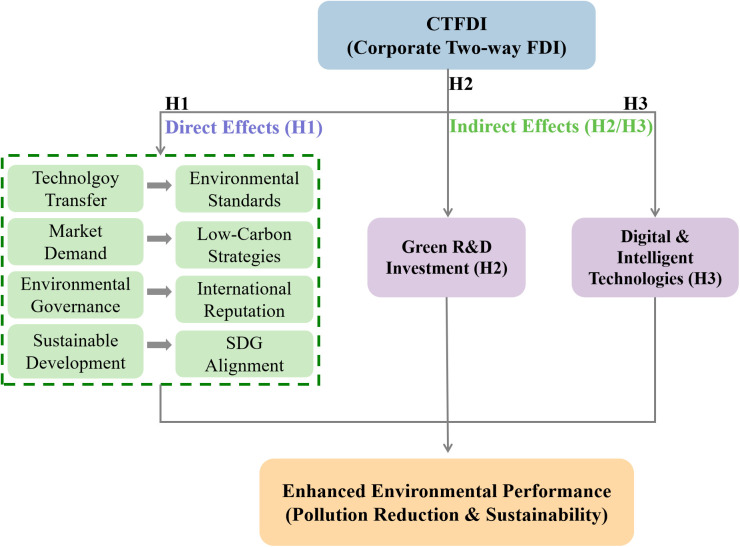
Conceptual framework illustrating the impact mechanisms of CTFDI on enhanced environmental performance.

To provide a clear overview of the theoretical logic and research hypotheses, Fig 1 presents the conceptual framework of this study. This framework illustrates how CTFDI affects corporate environmental performance through both direct and indirect mechanisms, integrating technology transfer, market demand alignment, environmental governance, sustainable development goals (SDGs), green R&D investment, and digital/intelligent technologies.

### 2.1. CTFDI and environmental pollution

#### 2.1.1. Technology transfer and environmental standards.

Developed countries have established stringent environmental regulatory frameworks, exemplified by standards such as International Organization for Standardization (ISO) 14001 and the EU’s Eco-Management and Audit Scheme (EMAS), which compel corporations to adopt advanced environmental technologies and management practices to meet market entry requirements [31]. Empirical evidence supports the pollution halo hypothesis, indicating that CTFDI facilitates the transfer of cleaner technologies from developed to developing countries and promotes environmental upgrading through enhanced R&D investment and technological innovation [32–34]. Furthermore, according to neo-institutional theory, cross-border investment exposes corporations to external pressures that drive conformity with host-country institutional norms, including those governing environmental performance. This dynamic is often described as “output environmentalism” whereby multinational corporations transfer advanced environmental technologies and management practices to host economies, potentially raising local environmental standards [35]. In line with this mechanism, real-world evidence shows that corporations exposed to strict foreign regulations often adopt cleaner production processes, upgrade waste treatment facilities, and pursue ISO 14001 certification, which has been linked to measurable reductions in carbon and other pollutant emissions [36]. Consequently, technology transfer and innovation facilitate corporate compliance with stringent environmental standards and reinforce their competitiveness in international markets.

#### 2.1.2. Market demand and low-carbon strategies.

As environmental awareness continues to rise, an increasing number of consumers are willing to pay a premium for low-carbon products [37]. CTFDI allows corporations to gain access to low-carbon technologies and adapt their production strategies in response, ultimately contributing to the reduction of pollution. In line with the Porter hypothesis, such investments may simultaneously strengthen a corporate’s market position and promote the development of environmentally friendly product lines. Increased awareness of environmental regulations in host countries further incentivizes corporations to prioritize green innovation, aligning with SDGs and addressing global demands for more stringent environmental governance [38].

#### 2.1.3. Environmental governance and international reputation.

CTFDI often harmonizes environmental practices across both corporate headquarters and overseas subsidiaries, rather than exploiting regulatory gaps in countries with more lenient environmental standards [39]. This approach reflects the pollution halo hypothesis, as it suggests that corporations engaged in CTFDI are more likely to internalize rigorous environmental standards and align their global operations with international environmental norms in response to regulatory and societal legitimacy pressures. Consequently, such corporations tend to place greater emphasis on public image, which in turn motivates improvements in environmental, social, and governance (ESG) performance [40]. A strong ESG profile enhances international reputation and drives continuous progress in environmental governance.

#### 2.1.4. CTFDI and SDGs.

This study posits that engagement in CTFDI enhances corporate environmental performance by reinforcing strategic orientation toward global sustainability norms. Through international investment activities, corporates become more receptive to external institutional pressures and increasingly attentive to their international reputation. In particular, alignment with the SDGs motivates corporates to adopt environmental certifications such as ISO 14001 and EMAS, which strengthen corporate credibility while mitigating compliance risks and long-term regulatory costs [39]. At the same time, in the pursuit of international competitiveness, corporations tend to increase investments in environmental protection. These efforts contribute to lower pollutant emissions and improved environmental performance [41]. This process reflects both the pollution halo hypothesis and environmental regulation theory, indicating that international investment can support corporate growth while promoting pollution reduction through technological upgrading and coordinated environmental governance.

The empirical analysis indicates that CTFDI contributes to improved environmental performance by facilitating the integration of advanced technologies, enhancing alignment with market expectations, reinforcing internal governance, and promoting compliance with global institutional norms [39,41]. These mechanisms are consistent with the pollution halo hypothesis, which highlights the beneficial spillover effects of foreign investment, as well as with institutional theory, which emphasizes how corporations adapt to international regulatory and normative pressures. Based on this theoretical foundation and empirical rationale, this study proposes Hypothesis 1.

H1. CTFDI has the potential to mitigate corporate environmental pollution.

### 2.2. Two-way FDI behavior and green R&D investment

Under the RBV, green innovation capability is regarded as a critical internal resource that enables corporations to achieve a sustainable competitive advantage. CTFDI enhances a corporation’s ability to access and integrate global innovation resources, thereby strengthening green R&D capacity and improving environmental performance. First, corporations engaged in CTFDI are more likely to adopt green development strategies, increase investment in energy-saving and emission-reducing technologies, and improve internal environmental governance [27,29]. These strategic efforts constitute valuable, rare, and inimitable internal capabilities that support long-term sustainability. Second, CTFDI facilitates higher levels of green R&D expenditure and accelerates technological advancement, which are key drivers in reducing environmental pollution [32]. Green technologies represent essential resources for value creation and sustainable growth [42]. Compared to corporations operating solely within domestic markets, those engaged in CTFDI benefit from cross-border knowledge flows and greater exposure to foreign innovation ecosystems, which leads to higher R&D intensity and greater allocation of revenue to innovation activities [43,44]. Third, as CTFDI corporations align their long-term goals with the SDGs, they are more likely to scale up sustainable innovation capabilities. By pursuing environmental certifications and cultivating international reputational capital, corporations strengthen their long-term commitment to green R&D, which in turn lowers compliance costs and enhances global competitiveness [45]. In summary, CTFDI strengthens corporate green innovation by mobilizing and integrating global and internal resources. Based on this logic, this study proposes Hypothesis 2.

H2. CTFDI is positively associated with green R&D investment and negatively associated with corporate environmental pollution.

### 2.3. Application of digital and intelligent technologies

The RBV also highlights the strategic value of digital and intelligent technologies as critical resources for achieving environmental transformation. Corporations engaged in CTFDI are more capable of acquiring, integrating, and deploying such technologies due to their international orientation and access to advanced innovation networks. CTFDI enables corporations to access cutting-edge digital tools from foreign markets, which help reduce information asymmetries, enhance operational efficiency, and strengthen environmental governance [46].

In this study, corporate environmental pollution is primarily reflected in industrial wastewater and industrial waste gas. Digital and intelligent technologies improve environmental performance by enabling real-time monitoring, predictive maintenance, and process optimization for these specific pollutants. For instance, intelligent water-treatment systems can effectively reduce chemical oxygen demand (COD) and ammonia nitrogen (AN) emissions, while digital process control and automated exhaust-treatment equipment lower waste gas emissions in energy-intensive manufacturing sectors such as chemicals and metallurgy. Lighter manufacturing sectors, such as electronics and textiles, benefit more from real-time monitoring and compliance management than from direct process optimization. In the context of CTFDI, these technologies are deployed both at overseas subsidiaries and domestic plants, forming an integrated environmental management system where international standards and digital monitoring directly drive reductions in wastewater and waste gas emissions.

As globally distributed organizations, these corporations leverage international partnerships to support intelligent coordination, network-based decision-making, and digital production upgrades [47]. Moreover, CTFDI often exposes corporations to host-country regulatory environments with stricter environmental standards, thereby incentivizing the adoption of advanced digital technologies for pollution monitoring, energy efficiency, and production optimization. Intelligent systems play a key role in automating waste treatment, enabling predictive maintenance, and facilitating low-carbon transition [48].

Guided by RBV, these digital and intelligent capabilities are considered strategic assets that help integrate sustainability into core operational processes. Therefore, this study puts forward Hypothesis 3.

**H3.** CTFDI is expected to enhance the application of digital and intelligent technologies, which will subsequently lead to a reduction in environmental pollution.

## 3. Data and methodology

### 3.1. Variables definitions and descriptions

#### 3.1.1. Dependent variable.

Environmental Pollution Management Level (TEP). Pollutant emissions at the corporate level reflect the degree of environmental pollution control, with emissions primarily comprising industrial wastewater (AP) and industrial waste gas (WP). Specifically, COD and AN represent key indicators of wastewater pollution, while sulfur dioxide (SO_2_) and nitrogen oxides (NO_X_) are typical indicators of air pollution. In this study, the emissions of these four pollutants are converted into a standardized measure of pollution equivalents based on the pollution equivalent values defined in the Measures for the Administration of Sewage Charge Collection Standards. The detailed calculation procedure is provided in Appendix A. As the emission data include zero values, directly applying the logarithmic transformation would result in truncation issues. To address this, the corporate emissions variable is log-transformed after adding one to the original value.

#### 3.1.2. Core explanatory variable.

CTFDI refers to the simultaneous presence of FDI and ODI within the same fiscal year. Following the approach of Yang et al. (2023) [49], a corporation is defined as engaging in CTFDI if it simultaneously exhibits both FDI and ODI activities, as detailed in Table 1. The identification of FDI behavior draws on the method proposed by Huang et al. (2019) [50]. A listed corporation is classified as engaging in FDI, with FDI coded as 1, if its equity structure includes foreign promoter shares, outstanding B-shares, outstanding H-shares, or other foreign outstanding shares. Otherwise, FDI = 0. Data on ODI behavior are obtained from the CSMAR database, which provides information on overseas business revenues, overseas branches, overseas transaction data, and statistical reports of overseas affiliates. These data are cross-verified with the Directory of Outward Investment Corporations published by the Ministry of Commerce of China. A corporation is considered to engage in ODI (i.e., ODI = 1) if it is listed in both data sources; otherwise, ODI = 0. Accordingly, a corporation is identified as engaging in two-way FDI behavior (CTFDI = 1) if both FDI = 1 and ODI = 1; otherwise, CTFDI = 0.

**Table 1:** Research Setting of CTFDI.

Pre-conditions		Results
corporate FDI Behavior	corporate ODI Behavior	CTFDI
FDI = 1	ODI = 1	CTFDI = 1
FDI = 1	ODI = 0	CTFDI = 0
FDI = 0	ODI = 1	CTFDI = 0
FDI = 0	ODI = 0	CTFDI = 0

#### 3.1.3. Mechanism variables.

Green R&D Investment. Green R&D investment is proxied by the number of R&D personnel (Rdpeo) and total R&D expenditure (Rdval). In addition, this study includes the number of green patent applications (Greenpats) and the number of green patent authorizations (Greenpatau) to further capture the outcomes of green innovation efforts. Higher values of these indicators suggest that a corporation is more actively engaging in innovation that can lead to lower emissions of wastewater and waste gas. All variables are expressed in natural logarithmic form to normalize the distribution and reduce heteroskedasticity.

Application of Digital and Intelligent Technologies. This dimension is measured by two variables, namely digital transformation speed (Digibs) and robot penetration (Robot). The digital transformation speed is calculated based on the frequency of digital-related keywords appearing in the annual reports of listed firms. Specifically, following Zhao et al. (2024) [51], this study identifies digital transformation keywords and constructs a word frequency index (WF). Based on the approach developed by Sun et al. (2024) [52], the WF index is then used to compute the speed of digital transformation (Digibs). A detailed description of Digibs and the WF index can be found in Appendix B.

Robot Penetration. From the perspective of robot penetration, Acemoglu and Restrepo (2020) [53] employed a general equilibrium model to examine the impact of robotics on regional labor markets in the United States. Based on their findings, they developed a regional-level measure of robot penetration using a “Bartik-like” approach, similar to the Bartik instrument [54,55]. Drawing on this approach, the present study constructs a firm-level robot penetration indicator for Chinese listed firms. The methodology consists of three steps. First, industry-level robot penetration rates are calculated based on industrial robot usage data. Second, these industry-level indicators are mapped to firm-level indicators according to each corporation’s primary industry classification. Third, United States industry-level robot usage data are applied to estimate robot penetration at the corporate level in China, following the spirit of the Bartik-like instrument approach. This method allows for an exogenous measure of automation exposure by leveraging variation in robot adoption intensity across industries, thereby mitigating endogeneity concerns.

#### 3.1.4. Control variables.

Following Chen et al. (2021) [56], Zhou et al. (2022) [57], and Xiang et al. (2022) [58], this study includes control variables, which consist of the year-end total asset growth rate (Asset), cash flow ratio (Cashflow), Tobin’s Q value (Tobinq), intangible asset ratio (Itang), years since listing (Age), and board size (Boardsize). Specifically, the year-end total asset growth rate is calculated as the ratio of the increase in total assets at year-end to the total assets at the beginning of the year, reflecting corporate growth, operational dynamics, and scale expansion. The cash flow ratio is defined as net cash flow from operating activities divided by current liabilities at the end of the period, indicating the corporation’s liquidity and ability to repay short-term debts. Tobin’s Q is measured as the ratio of a corporation’s market value to the replacement cost of its assets, serving as an indicator of market valuation. The intangible asset ratio is calculated as the ratio of net intangible assets to total assets, and reflects a corporation’s capacity for resource reconfiguration and innovation. The variable Age represents the number of years since a corporation’s initial listing, computed as the current fiscal year minus the listing year plus one. Boardsize refers to the number of members on the board of directors.

### 3.2. Data method and descriptions

This study utilizes a balanced panel dataset comprising 43,410 observations from 2,894 A-share listed firms in China over the period 2008–2022. As one of the largest stock markets globally, China’s capital market includes a broad spectrum of listed firms spanning diverse industries. Focusing on A-share listed firms enables a representative and comprehensive analysis of corporate economic behavior and provides valuable insights into the broader trajectory of China’s economic development. The study period begins in 2008, following the implementation of China’s new accounting standards in 2007, which ensures consistency and comparability in financial data. To improve data quality and avoid extreme bias, the sample excludes four types of corporations, including financial and insurance corporations, ST and PT corporations under special regulatory treatment, those with a gearing ratio exceeding 1, and those with missing values for key variables. Data on corporate pollutant emissions were obtained from the National Tax Survey, administered by tax authorities using a stratified random sampling method. This dataset provides detailed information on firm-level energy consumption and pollution emissions. Data for other firm-level variables were collected from annual report disclosures and the CSMAR database, which offers comprehensive information on the financial, operational, and governance characteristics of listed firms in China. Variable definitions are provided in Table 2.

**Table 2:** Definition table for baseline regression variables.

Variable abbreviation	Variable Meaning	measurements	Data Sources
**TEP**	Environmental pollution management level	See section 3.1.1.	Data from the National Tax Survey
**AP**	Air pollution management level	See section 3.1.1.	Data from the National Tax Survey
**WP**	Water pollution management level	See section 3.1.1.	Data from the National Tax Survey
**CTFDI**	Two-way FDI Behavior	See section 3.1.2.	Annual report disclosures and CSMAR database
**Asset**	Year-end total asset growth rate	Ratio of increase in total assets at the end of the year to total assets at the beginning of the year	CSMAR database
**Cashflow**	Cash flow ratios	Net cash flows from operating activities to current liabilities at the end of the period	CSMAR database
**Tobinq**	Tobin’s Q-value	Market value of the corporate as a percentage of the replacement cost of the asset	CSMAR database
**Itang**	Intangible assets ratio	Net intangible assets as a percentage of total assets	CSMAR database
**Age**	Number of years as a listed corporate	Fiscal year less date of incorporation plus 1	CSMAR database
**Boardsize**	Board size	Number of board members	CSMAR database

Based on the descriptive statistics presented in Table 3, the mean value of CTFDI is 0.1515 with a standard deviation of 0.3585, indicating that approximately 15.15% of the sample corporations engage in two-way FDI. This reflects a notable presence of CTFDI behavior among A-share listed firms in China. The mean value of TEP is 0.1440, with a maximum of 0.1529 and a minimum of 0.1257. This variation in environmental pollution management levels suggests significant heterogeneity in pollution emissions across listed firms. These differences underscore the necessity of further investigating the potential causal relationship between international investment behavior and corporate environmental performance.

**Table 3:** Descriptive statistics.

Variable	N	Mean	Median	Min	Max	Sd
**TEP**	43,410	0.1440	0.1457	0.1257	0.1529	0.0053
**AP**	43,410	0.1522	0.1538	0.1344	0.1613	0.0054
**WP**	43,410	0.1382	0.1397	0.1173	0.1489	0.0056
**CTFDI**	43,410	0.1515	0.0000	0.0000	1.0000	0.3585
**Cashflow**	43,410	0.1764	0.1368	−0.0598	1.0000	0.1398
**Tobinq**	43,410	2.8343	1.6470	0.6245	14810.3060	97.6498
**Itang**	43,410	0.0483	0.0342	0.0000	0.9383	0.0603
**Age**	43,410	15.4730	15.0000	0.0000	64.0000	7.1664
**Boardsize**	43,410	8.2767	8.0000	0.0000	18.0000	1.4802

### 3.3. Empirical model

To ensure the validity of the empirical model, a series of diagnostic tests were conducted prior to regression estimation. First, the variance inflation factor (VIF) test shows that all variables have VIF values below 1.05, indicating no evidence of multicollinearity. Second, Pesaran’s cross-sectional dependence (CD) test strongly rejects the null hypothesis of cross-sectional independence (P = 0.000), suggesting significant cross-sectional dependence across sample corporations [59]. To address this issue, the study adopts a multiple fixed-effects OLS regression model controlling for province, industry, and year, and implements both cluster-robust and Driscoll–Kraay standard errors. Third, panel unit root tests using the Fisher-ADF and Levin–Lin–Chu (LLC) methods confirm that the main variables are stationary, with all p-values below 0.01. Finally, the Hausman test (P = 0.000) rejects the null hypothesis in favor of the fixed-effects model, supporting the use of multiple fixed-effects to control for unobserved heterogeneity and ensure consistent parameter estimation.

Based on these diagnostic results, the relationship between CTFDI and corporate environmental pollution is estimated using a multiple fixed-effects OLS model, as specified in equation (1).


$$TEP_{ipt}=\beta_{\text{0}}+\beta_{\text{1}}\times CTFDI_{ipt}+\lambda X_{ipt}+\theta_{i}+\eta_{p}+\delta_{t}+\varepsilon_{ipt}$$ (1)

In Equation (1), i, p, and t represent industry, province, and year, respectively. The explanatory variable TEP denotes corporate environmental pollution. The core explanatory variable IOFDI denotes corporate two-way FDI, which is CTFDI. X denotes control variables, which are total asset growth rate (Asset), cash flow ratio (Cashflowr), Tobin’s Q value (Tobinq), intangible asset share (Itang), corporations’ years of establishment (Age), and board of directors size (Boardsize). θ denotes industry fixed-effects, η denotes province fixed-effects, and δ denotes year fixed-effects. ε is a random disturbance term. The model is estimated by adjusting for clustering of standard errors at the corporate level. The coefficient to be estimated, $\beta_{\text{1}}$, reflects the extent of the impact of CTFDI on environmental pollution. Furthermore, to test H2 and H3, this study draws on Jiang (2022) [60] and replaces the explanatory variables with mediating variables to explore the influence mechanism of CTFDI and environmental pollution. All other variable settings are consistent with Equation (1).

## 4. Empirical results

### 4.1. Baseline results

Table 4 presents the results of the baseline regression examining the impact of CTFDI on environmental pollution management. Column (1) reports the results without any control variables. The coefficient of CTFDI is significantly negative, indicating that CTFDI behavior is associated with a reduction in overall pollution emissions and an improvement in environmental pollution management. Specifically, compared to corporations that do not engage in CTFDI, those that do exhibit a 0.17% lower level of pollution emissions. In Column (2), after incorporating the full set of control variables, the coefficient of CTFDI remains significantly negative at the 1% level, with an estimated value of −0.0015. This suggests that, holding other factors constant, a one-unit increase in CTFDI corresponds to a 0.15% reduction in pollution emissions on average. These results provide strong empirical support for H1. The consistency across model specifications underscores the robustness of the relationship and suggests that CTFDI plays a significant role in reducing environmental pollution through mechanisms such as industrial coordination and technology spillovers. Overall, the findings highlight the importance of integrating two-way FDI into international investment strategies aimed at achieving environmental sustainability. The evidence reinforces the view that CTFDI contributes to lower corporate pollutant emissions, thereby enhancing our understanding of how international economic activities influence environmental outcomes.

**Table 4:** Baseline regression results.

Variables	(1)	(2)
**CTFDI**	−0.0017***	−0.0015***
	(−24.18)	(−19.90)
**Cashflowr**		−0.0014***
		(−4.55)
**Tobinq**		−0.0000***
		(−11.65)
**Itang**		0.0016**
		(2.45)
**Age**		−0.0002***
		(−18.43)
**Boardsize**		−0.0003***
		(−12.07)
**Constant**	0.1443***	0.1501***
	(2,711.36)	(553.20)
**Observations**	43,410	43,410
**R-squared**	0.492	0.538
**Industry FE**	Yes	Yes
**Province FE**	Yes	Yes
**Year FE**	Yes	Yes

***, **, * indicate significance levels of 1%, 5%, and 10%, respectively, Robust t-statistics in parentheses, the same applies to the following tables.

### 4.2. Endogenous test

Prior studies have demonstrated a strong association between CTFDI and trade activity. Specifically, Goh and Wong (2014) [61] and Tham et al. (2018) [62] established the complementarity between CTFDI and corporate import-export behavior, while Li et al. (2021) [63] further showed that two-way FDI significantly contributes to export sophistication. These findings suggest a close linkage between CTFDI and trade activities, as captured by the IMEX variable (defined in Table 5). However, trade activity (IMEX) is not directly related to corporate pollution emissions, satisfying the exclusion restriction required for valid instrumental variables. To address potential endogeneity in the estimation of the relationship between CTFDI and pollution emissions, this study employs IMEX as an instrumental variable (IV) for CTFDI. The IV estimation results are reported in Table 5, Columns (1) and (2). This approach helps to mitigate concerns about reverse causality and omitted variable bias, thereby enabling a more credible identification of the causal impact of CTFDI on environmental pollution. The results obtained from the IV estimation further reinforce the baseline findings, suggesting that CTFDI exerts a statistically significant and negative effect on corporate pollution emissions. Overall, the use of trade activity as an instrument allows this study to isolate the exogenous variation in CTFDI and offers more robust evidence for its pollution-reducing effects. These findings contribute to the literature on sustainable internationalization strategies and underscore the potential of CTFDI as a pathway toward improved environmental performance.

**Table 5:** Results of IV-2SLS regression.

Variables	(1) CTFDI	(2) TEP
**IMEX**	0.1082***	
	(7.89)	
**CTFDI**		−0.0269***
		(−7.83)
**Asset**	0.0038	0.0008**
	(0.33)	(2.57)
**Cashflowr**	0.0495*	−0.0004
	(1.80)	(−0.52)
**Tobinq**	0.0000***	0.0000**
	(5.80)	(2.43)
**Itang**	0.1214	0.0041
	(1.19)	(1.52)
**Age**	0.0005	−0.0001***
	(0.48)	(−5.08)
**Boardsize**	0.0213***	0.0003**
	(5.50)	(2.15)
**Kleibergen-Paap rk LM statistic**	60.093	
**Cragg-Donald Wald F statistic**	805.206	
**Observations**	43,410	43,410
**Industry FE**	Yes	Yes
**Province FE**	Yes	Yes
**Year FE**	Yes	Yes

Table 5 presents the results of the two-stage least squares (2SLS) regression using IMEX as an instrumental variable for CTFDI. Column (1) reports the first-stage regression results, where the coefficient on IMEX is significantly positive. This indicates a strong and statistically meaningful association between trade activity and CTFDI, thereby supporting the relevance condition of the instrumental variable. The strong correlation confirms that corporate import-export behavior is a valid predictor of CTFDI engagement. Furthermore, weak instrument diagnostics indicate that the instrument is not weak, and the first-stage F-statistic exceeds the conventional thresholds, thereby validating the strength of IMEX as an instrument. Additional tests also support the exogeneity assumption, confirming that IMEX is uncorrelated with the error term in the second-stage regression. These results collectively affirm the validity and reliability of the IV strategy employed in this study. Column (2) reports the second-stage regression estimates. The coefficient on the fitted value of CTFDI remains significantly negative, consistent with the results from the baseline OLS regression. This finding provides robust evidence that CTFDI contributes to the reduction of corporate pollutant emissions. The application of the IV-2SLS method helps mitigate concerns related to endogeneity, such as reverse causality and omitted variable bias, thereby reinforcing the causal interpretation of the relationship between CTFDI and environmental pollution management. Overall, the consistent and significant results across both stages of the IV estimation reinforce the conclusion that CTFDI is an effective channel for promoting environmentally sustainable practices and advancing green transformation in corporate operations.

### 4.3. Robustness stability test

Table 6 reports the robustness regression results evaluating the effect of CTFDI on corporate environmental pollution management. To ensure the reliability of the baseline findings, three robustness strategies are employed, namely variable substitution, alternative fixed-effects specifications, and sample restriction. Columns (1) and (2) report the results using disaggregated pollutant measures by substituting the dependent variable with industrial wastewater emissions and industrial exhaust emissions, respectively. The coefficients on CTFDI remain significantly negative, indicating that two-way FDI behavior is effective in reducing both types of emissions. Columns (3) and (4) test the sensitivity of the results to different fixed-effects specifications. In column (3), industry-year and province fixed-effects are included, while column (4) controls for city, industry, and year fixed-effects. The estimated coefficients for CTFDI are −0.0014 and −0.0016, respectively, both statistically significant at the 1% level. These results confirm that the findings are not driven by omitted regional or temporal heterogeneity. Column (5) addresses potential distortions caused by the COVID-19 pandemic by excluding observations from 2020 onwards. The estimated coefficient of −0.0018 for CTFDI remains significantly negative, further confirming the robustness of the results [64]. Overall, the results across all robustness checks are consistent with those of the baseline regression, providing strong evidence that the empirical findings are stable and reliable.

**Table 6:** Results of robustness analysis.

Variables	(1) WP	(2) AP	(3) TEP	(4) TEP	(5) TEP
**CTFDI**	−0.0015***	−0.0015***	−0.0014***	−0.0016***	−0.0018***
	(−18.67)	(−19.83)	(−19.32)	(−19.79)	(−19.85)
**Cashflowr**	−0.0015***	−0.0014***	−0.0017***	−0.0014***	−0.0017***
	(−4.46)	(−4.62)	(−5.29)	(−4.56)	(−4.92)
**Tobin**	−0.0000***	−0.0000***	−0.0000***	−0.0000***	−0.0000***
	(−12.75)	(−9.07)	(−5.30)	(−11.67)	(−10.42)
**Itang**	0.0017**	0.0017**	0.0013*	0.0017**	0.0017**
	(2.33)	(2.48)	(1.91)	(2.54)	(2.25)
**Age**	−0.0002***	−0.0002***	−0.0002***	−0.0002***	−0.0002***
	(−18.19)	(−18.34)	(−18.32)	(−17.67)	(−18.49)
**Boardsize**	−0.0003***	−0.0003***	−0.0003***	−0.0003***	−0.0004***
	(−11.83)	(−11.92)	(−11.34)	(−12.21)	(−12.02)
**Constant**	0.1444***	0.1583***	0.1501***	0.1500***	0.1499***
	(510.27)	(578.49)	(546.19)	(550.11)	(497.40)
**Observations**	43,410	43,410	43,380	43,410	37,622
**R-squared**	0.495	0.528	0.576	0.553	0.472
**Industry FE**	Yes	Yes	No	Yes	Yes
**Province FE**	Yes	Yes	Yes	No	Yes
**Year FE**	Yes	Yes	No	Yes	Yes
**Industry-Year FE**	No	No	Yes	No	No
**City FE**	No	No	No	Yes	No

### 4.4. Heterogeneity test

Table 7 reports the results of the heterogeneity analysis based on corporate ownership, industry type, and regional distribution. Columns (1) and (2) present the regression results of CTFDI on the level of environmental pollution governance for state-owned enterprises (SOEs) and non-state-owned enterprises (non-SOEs), respectively. The results demonstrate that the pollution-mitigating effect of CTFDI is substantially stronger for non-SOEs (−0.0016, significant at the 1% level) than for SOEs (−0.0007, significant at the 1% level). The relatively limited responsiveness of SOEs may stem from their close alignment with government mandates, which can reduce their sensitivity to market-driven incentives embedded in CTFDI behavior. In contrast, non-SOEs are more responsive to market forces and environmental standards, allowing them to better leverage the spatial spillovers and total factor productivity gains associated with CTFDI. This finding underscores the greater potential of non-SOEs to capitalize on the environmental sustainability benefits of CTFDI, especially in private-sector contexts. Columns (3) and (4) show the regression results for corporations in heavily polluting industries and non-heavily polluting industries, respectively. The results show that CTFDI mitigates pollution more effectively in non-heavily polluting industries (−0.0016, significant at the 1% level) than in heavily polluting industries (0.0000, not significant), implying that international investment facilitates environmental upgrading primarily in sectors with less rigid pollution structures. Columns (5) and (6) report results based on regional heterogeneity, specificall y comparing corporations located east and west of the Hu Line. CTFDI demonstrates a stronger pollution-reducing effect in the eastern region (−0.0015,significant at the 1% level) than in the western region (−0.0006, not significant), reflecting the east’s greater economic openness, stronger institutions, and more advanced technological infrastructure.These regional differences further highlight the contextual factors influencing the effectiveness of CTFDI in promoting environmental governance.

**Table 7:** Heterogeneity analysis results.

Variables	(1) SOEs	(2) non-SOEs	(3) Heavily Polluted Industry	(4) Non-heavily Polluted Industry	(5) East of Hu Line	(6) West of Hu Line
**CTFDI**	−0.0007***	−0.0016***	0.0000	−0.0016***	−0.0015***	−0.0006
	(−7.05)	(−16.88)	(0.66)	(−17.52)	(−19.98)	(−1.16)
**Cashflowr**	−0.0002	−0.0029***	0.0011***	−0.0017***	−0.0015***	0.0002
	(−0.55)	(−7.74)	(8.23)	(−5.05)	(−4.62)	(0.15)
**Tobin**	−0.0000***	−0.0000**	−0.0000	−0.0000***	−0.0000***	−0.0001
	(−6.45)	(−2.51)	(−1.65)	(−12.69)	(−11.77)	(−1.54)
**Itang**	0.0006	0.0006	−0.0002	0.0008	0.0016**	0.0030
	(1.10)	(0.50)	(−0.66)	(1.09)	(2.34)	(1.01)
**Age**	−0.0001***	−0.0002***	−0.0000***	−0.0002***	−0.0002***	−0.0004***
	(−6.90)	(−12.88)	(−5.44)	(−16.28)	(−18.09)	(−6.29)
**Boardsize**	−0.0001***	−0.0003***	0.0000	−0.0003***	−0.0003***	−0.0001
	(−3.29)	(−7.69)	(0.73)	(−11.02)	(−12.20)	(−0.60)
**Constant**	0.1452***	0.1506***	0.1424***	0.1504***	0.1502***	0.1491***
	(330.34)	(403.47)	(1,426.46)	(519.31)	(547.16)	(119.42)
**Observations**	14,028	29,382	8,639	34,771	42,240	1,170
**R-squared**	0.819	0.433	0.961	0.507	0.533	0.784
**Industry FE**	Yes	Yes	Yes	Yes	Yes	Yes
**Province FE**	Yes	Yes	Yes	Yes	Yes	Yes
**Year FE**	Yes	Yes	Yes	Yes	Yes	Yes

### 4.5. Mechanism analysis

To test the mediating mechanisms proposed in Hypotheses H2 and H3, this study examines two key channels, namely green R&D investment and the application of digital and intelligent technologies. These mediators are expected to capture how CTFDI indirectly improves corporate environmental performance. Specifically, H2 posits that CTFDI enhances corporate green innovation efforts, thereby reducing environmental pollution, while H3 proposes that CTFDI facilitates the adoption of digital and intelligent technologies, which in turn strengthen pollution control and resource efficiency. The mediating variables are incorporated into the empirical model as *MA*_*ipt*_ in Equation (2) to examine whether CTFDI indirectly influences environmental pollution via green R&D investment (H2) and the application of digital and intelligent technologies (H3). This structure provides a clearer understanding of the hypothesized mechanisms and their role in corporate environmental performance.


$${MA}_{ipt}=\beta_{\text{0}}+\beta_{\text{1}}\times CTFDI_{ipt}+\lambda X_{ipt}+\theta_{i}+\eta_{p}+\delta_{t}+\varepsilon_{ipt}$$ (2)

#### 4.5.1. Green R&D investment.

Green R&D investment is measured using four indicators, namely the number of R&D personnel (Rdpeo), R&D expenditure (Rdval), the number of green patent applications (Greenpats), and the number of green patent authorizations (Greenpatau). All variables are transformed using the natural logarithm to ensure normality and comparability. Columns (1) through (4) of Table 8 report the regression results assessing the impact of CTFDI on corporate green R&D investment. Across all four models, the coefficients of CTFDI are significantly positive at the 1% level. These results indicate that CTFDI exhibit significantly higher levels of R&D personnel, R&D spending, and both green patent applications and authorizations. The findings suggest that CTFDI promotes the expansion of green R&D efforts, thereby strengthening corporate innovation capacity and environmental competitiveness. By enhancing green R&D investment, CTFDI enables corporations to reinforce their ownership advantages and generate positive externalities, ultimately contributing to pollution reduction. In summary, the results presented in Table 8 demonstrate a robust positive association between CTFDI and green R&D investment, confirming that CTFDI plays a pivotal role in fostering technological innovation for environmental sustainability. Based on the theoretical framework outlined in the mechanism section, these findings support the conclusion that CTFDI contributes to improved environmental pollution management by stimulating green R&D activities. Therefore, H2 is supported.

**Table 8:** Mechanism analysis results.

Variables	(1) lnRdpeo	(2) lnRdval	(3) lnGreenpats	(4) lnGreenpatau	(5) digibs	(6) robot
**CTFDI**	0.2609***	0.3745***	0.2337***	0.1967***	0.0066*	1.0376***
	(4.36)	(5.77)	(4.88)	(4.72)	(1.78)	(14.95)
**Cashflowr**	−0.8192***	0.1446***	−0.6676***	−0.6082***	0.0777***	−1.5002***
	(−5.38)	(8.79)	(−8.08)	(−8.98)	(4.47)	(−7.40)
**Tobinq**	−0.1310***	−0.6750***	0.0001***	0.0001***	−0.0000***	0.0003***
	(−6.87)	(−5.09)	(3.65)	(4.86)	(−8.04)	(6.83)
**Itang**	0.2509	−0.1134***	0.1449	0.2226	0.0416	−1.0941**
	(0.57)	(−5.87)	(0.60)	(1.05)	(1.31)	(−2.37)
**Age**	0.0025	−0.7493	−0.0008	−0.0014	0.0000	0.1280***
	(0.57)	(−1.39)	(−0.25)	(−0.51)	(0.01)	(18.02)
**Boardsize**	0.1181***	−0.0079	0.0984***	0.0765***	0.0015	0.1719***
	(7.46)	(−1.59)	(8.24)	(7.36)	(1.32)	(8.62)
**Constant**	4.8479***	16.9184***	0.0993	0.0964	0.0468***	1.2291***
	(28.57)	(98.10)	(0.84)	(0.94)	(4.16)	(6.38)
**Observations**	10,400	16,788	30,166	30,166	29,102	43,410
**R-squared**	0.280	0.328	0.271	0.272	0.032	0.281
**Industry FE**	Yes	Yes	Yes	Yes	Yes	Yes
**Province FE**	Yes	Yes	Yes	Yes	Yes	Yes
**Year FE**	Yes	Yes	Yes	Yes	Yes	Yes

#### 4.5.2. The application of digital and intelligent technologies.

The application of digital and intelligent technologies in this study is measured using two indicators, namely digital transformation speed (Digibs) and industrial robot penetration (Robot). Column (5) of Table 8 shows that CTFDI has a significantly positive effect on digital transformation speed, suggesting that corporations engaged in CTFDI are more likely to accelerate their digital upgrading. From the perspective of robot penetration, the coefficient of CTFDI in Column (6) is 1.0376 and statistically significant at the 1% level, indicating that CTFDI is associated with a higher adoption rate of industrial robots compared to corporations without such international investment activities. China’s industrial robot market has witnessed rapid development, attracting leading global manufacturers such as ABB (Sweden), KUKA (Germany), Fanuc and Yaskawa Electric (Japan), FUJIKOSHI (Japan), and COMAU (Italy). These corporations have established production and service facilities in key robotics industrial parks located in major cities such as Shanghai, Kunshan, Chongqing, and Qingdao. CTFDI contribute to this ecosystem by bringing in capital, technical expertise, quality standards, and management experience. These inputs generate “active technology spillovers,” including both “export learning effects” and “embedded learning effects,” which promote the diffusion of advanced production technologies and managerial practices among domestic corporations. Moreover, the high-quality requirements imposed by CTFDI increase the pressure on domestic corporations to pursue continuous technological upgrading. This dynamic fosters industrial upgrading and enhances the effectiveness of environmental pollution management. Theoretical and empirical findings jointly confirm that CTFDI plays a significant role in advancing corporate adoption of digital and intelligent technologies, as evidenced by higher levels of industrial robot penetration. These results offer robust support for H3.

## 5. Discussion

This study examines the environmental implications of CTFDI by assessing its impact on pollution reduction and environmental performance at the corporate level. The baseline regression results indicate that CTFDI significantly reduces emissions, with an estimated coefficient of −0.0015. Our firm-level results (−0.0015) are consistent with Tian et al. (2023) [27](−0.0019), despite differences in data granularity and methodological focus. While Tian et al. (2023) [27] capture industry-level spillovers, our micro-level analysis highlights the internal corporate mechanisms that produce similar environmental benefits, reinforcing the robustness of the relationship across scales. These results provide empirical support for the pollution halo hypothesis, which posits that international investment improves environmental outcomes by facilitating the diffusion of advanced technologies and environmental management standards. The observed enhancement in environmental performance is also consistent with the RBV, as CTFDI enables corporations to acquire strategic assets, including green innovation capacity and digital infrastructure, both of which contribute to building a sustainable competitive advantage. For clarity, a summary table of all hypotheses and their outcomes is provided in Appendix C, offering a concise overview of the empirical results.

This study addresses two critical gaps in the existing literature. First, while prior research has primarily examined the environmental effects of two-way FDI at the regional, sectoral, or national levels [28,29,65,66], this study focuses on the corporate level. By incorporating corporate heterogeneity in ownership, industry characteristics, and investment behavior, the study provides a more nuanced understanding of the mechanisms through which CTFDI influences environmental performance. This micro-level perspective aligns with institutional theory, which emphasizes how corporations adapt strategically to external pressures, including environmental regulations and global sustainability norms. Second, the study advances the literature by moving beyond correlation to explore the internal mechanisms through which CTFDI affects environmental performance. Specifically, it demonstrates that CTFDI enhances green R&D investment and accelerates the application of digital and intelligent technologies [32,42,43,45,47]. Green R&D directly reduces pollution through increased R&D staffing and expenditures, which result in new green patents and process innovations that lower COD. Digital and intelligent technologies provide an operational pathway. Real-time monitoring and predictive maintenance help detect and address emission sources early, while automated water-treatment and exhaust-control systems improve resource efficiency. For example, in energy-intensive sectors such as chemicals and metallurgy, digital process optimization lowers waste gas emissions, whereas in electronics and textiles, digital monitoring primarily strengthens compliance and reporting. These findings reinforce the RBV by illustrating how access to international innovation networks and strategic technological resources contributes to long-term environmental capability and corporate sustainability.

In conclusion, this study provides robust firm-level evidence that CTFDI plays a significant role in improving environmental performance by fostering green R&D and promoting the adoption of digital and intelligent technologies. The empirical findings offer strong support for the pollution halo hypothesis, showing that strategically coordinated international investment facilitates the transfer of environmentally beneficial technologies and practices. The study also reinforces the RBV by highlighting how corporations leverage global operations to build environmental capabilities that sustain long-term competitive advantage. By integrating corporate heterogeneity and drawing on institutional theory, this study further illustrates how CTFDI enables corporations to respond proactively to environmental regulations and international sustainability norms. Overall, the findings contribute to the ongoing debate between the pollution halo and pollution haven hypotheses, demonstrating that, under appropriate institutional and strategic conditions, CTFDI serves as a powerful mechanism for aligning economic globalization with environmental governance. These insights are especially relevant as China and other emerging economies pursue carbon neutrality and green transformation, offering meaningful implications for corporate strategy and environmental policymaking.

## 6. Conclusion, implications, limitations and future research

### 6.1. Conclusion

Using a balanced panel of 2,894 Chinese A-share listed corporations from 2008 to 2022, this study investigates the environmental implications of CTFDI. The results show that CTFDI significantly reduces emissions, providing corporate-level evidence for the pollution halo hypothesis. Mechanism analysis demonstrates that CTFDI promotes green R&D investment and accelerates the adoption of digital and intelligent technologies, which together enhance innovation capacity and strengthen environmental compliance. Heterogeneity analysis indicates that the effect is more evident in non-SOEs, non-heavily polluting industries, and corporations located in eastern regions. Overall, the findings suggest that CTFDI can serve as a potential pathway for aligning internationalization with environmental sustainability.

### 6.2. Implications

Theoretical implications. This study provides firm-level evidence for the pollution halo hypothesis, showing that CTFDI reduces emissions through green R&D and digitalization. This micro-level perspective complements prior regional and sectoral studies and helps explain how the pollution halo effect emerges within corporations. The findings also extend the RBV by demonstrating that international investment facilitates the development of environmental capabilities, thereby transforming sustainability from a compliance requirement into a source of competitive advantage.

Practical implications. For managers, the results suggest that integrating environmental objectives into FDI decisions can improve efficiency, ESG performance, and regulatory compliance. Corporations that direct CTFDI toward R&D and digital transformation achieve more substantial environmental gains, providing a practical pathway for firms in emerging economies facing similar pressures of globalization and green transition.

Policy implications. The evidence indicates that CTFDI may serve as a policy instrument for advancing green development. Governments can enhance its effectiveness by supporting green R&D, promoting digital adoption, and designing differentiated policies that prioritize non-SOEs and less-polluting sectors, while gradually facilitating transitions in more polluting industries. Such measures would help reconcile economic upgrading with environmental sustainability.

### 6.3. Limitations and future research

Several limitations should be acknowledged. First, the sample is restricted to listed firms, which may not capture the conditions of smaller or less regulated firms. Second, CTFDI is measured in binary terms, which may not reflect investment intensity or host-country heterogeneity. Third, while the study highlights green R&D and digital adoption as key channels, it does not fully capture organizational and governance processes that may influence environmental outcomes. Future research could employ continuous measures of CTFDI, expand the scope to unlisted or cross-national samples, and apply qualitative or mixed methods to deepen understanding of how corporations integrate environmental strategies into internationalization. These efforts would enhance both the explanatory depth and generalizability of the findings.

## Supporting information

S1 File Appendix. (DOCX)
