# Supplementary material for: Does China’s corporate two-way foreign direct investment mitigate environmental pollution?
Source: PLoS One. 2025 Oct 7;20(10):e0333935. doi: 10.1371/journal.pone.0333935 (PMC12503249; doi:10.1371/journal.pone.0333935)
Supplement: S1 File — (DOCX) [file pone.0333935.s001.docx]

Appendix A Pollution Emissions

At present, the data on pollution emissions of listed firms that are most commonly utilized are primarily drawn from the CSMAR database on environmental research of Chinese listed firms. While this data set provides insight into the resource consumption and pollution emission patterns of listed firms in China, it is limited by a relatively small sample size and a starting year of 2015. This restricts the temporal and spatial scope of the data, which in turn constrains the precision and breadth of the analytical approaches that can be employed. To increase the sample size as much as possible, this paper further combines the tax survey data of listed firms, refers to the information related to environmental pollution in the report, and expands the pollution emission data in terms of the number of individual corporations and the time span. The pollution emission data of listed manufacturing corporations comprises five principal pollutant indicators, namely chemical oxygen demand and ammonia nitrogen emissions for water pollution, and sulfur dioxide, nitrogen oxides, and smoke and dust emissions for air pollution. Given the lack of uniformity in the units of measurement across different types of emissions, this study employs the methodology of Su and Sheng (66) to quantify the comprehensive indicators of pollution emissions at the corporate level.

First, the raw data for the above pollutant indicators were standardized using the extreme value method.

|  | (A1) |
| --- | --- |

where denotes the emission of pollutant n per unit of output of corporate i in period t; max and min denote the maximum and minimum values of pollutant n emissions per unit of output of all corporations per year, respectively.

Second, the adjustment factor for pollutant n in corporate i is calculated as follows.

| $\omega_{nit}=\frac{rpol_{nit}}{\overline{rpol_{nt}}}$ | (A2) |
| --- | --- |

where $\overline{rpol_{nt}}$ denotes the national average of pollutant n emissions per unit of output for all corporations.

Thirdly, combining equations (A1) and (A2), a composite index of pollution emissions per unit of output of corporate i is obtained:

| $ei_{it}=\frac{1}{6}\sum_{n} (rpol_{nit}\times\omega_{nit})$ | (A3) |
| --- | --- |

The larger the value of ei, the higher the pollution emission intensity of the corporate.

## Appendix B Revised Text for Digital Transformation Speed

The digital transformation speed (Digibs) is calculated based on the frequency of digital‑related keywords appearing in the annual reports of listed firms, as this textual disclosure reflects the intensity and pace of digital strategy implementation. Specifically, following Zhao et al. (2024) [51], this study first develops a digital transformation keyword dictionary, which includes terms such as artificial intelligence, cloud computing, big data, blockchain, digital platform, and smart manufacturing. These keywords are identified from policy documents, industry reports, and prior literature on digitalization. Next, for each corporation *i* in year *t*, the word frequency (WF) index is computed as in Equation (B1). This index represents the normalized frequency of digital terms per 1,000 words, controlling for variations in report length.

| $\mathrm{WF}_{\mathrm{ipt}}=\frac{{Number of digital keywords in annual report}_{\mathrm{ipt}}}{{Total words in annual report}_{\mathrm{ipt}}}\times1000$ | (B1) |
| --- | --- |

Finally, following Du and Ma (2024) [52], the digital transformation speed is measured as the first-order difference in the WF index to capture the annual growth rate of digitalization disclosure, as in Equation (B2).

| ${Digibs}_{ipt}={(WF}_{ipt}-{WF}_{i,p,t-1})/{WF}_{i,p,t-1}$ | (B2) |
| --- | --- |

A higher value of *Digibs_ipt_* indicates that a corporation is accelerating its digital transformation efforts, which can improve operational efficiency, enhance real-time environmental monitoring, and facilitate pollution reduction. This measure aligns with the rationale that textual disclosure in annual reports is a reliable proxy for the intensity and dynamics of corporate digital transformation.

## Appendix C Summary of Hypotheses and Empirical Results

Table C1. Summary of Hypotheses and Empirical Results

| **Hypothesis** | **Description** | **Key Variables / Indicators** | **Key Finding** | **Supported** |
| --- | --- | --- | --- | --- |
| H1 | CTFDI mitigates corporate environmental pollution. | Dependent variable: Corporate environmental pollution (TEP), measured by standardized pollution equivalents of: ① Chemical Oxygen Demand (COD), ② Ammonia Nitrogen (NH₃‑N), ③ Sulfur Dioxide (SO₂), ④ Nitrogen Oxides (NOₓ). | Baseline regression shows that CTFDI significantly reduces emissions (β = ‑0.0015, p < 0.01), supporting the pollution halo hypothesis. | Yes |
| H2 | CTFDI enhances green R&D investment, thereby improving environmental performance. | Green R&D investment (Mediator): ① Number of R&D personnel (Rdpeo), ② R&D expenditure (Rdval), ③ Green patent applications (Greenpats), ④ Green patent authorizations (Greenpatau) (all ln‑transformed). | Mediation analysis shows that CTFDI significantly increases green R&D inputs and outputs, which in turn contribute to reduced emissions of wastewater and waste gas. | Yes |
| H3 | CTFDI promotes the application of digital and intelligent technologies, which indirectly reduces pollution. | Digital & intelligent technologies (Mediator): ① Digital transformation speed (Digibs) – calculated via word frequency (WF) index of digital‑related keywords in annual reports and first‑order difference year‑over‑year; ② Industrial robot penetration (Robot) – measured using a Bartik‑like approach combining industry‑level robot usage and U.S. adoption data for exogenous corporate‑level exposure. | Mediation analysis confirms that CTFDI accelerates digitalization and automation, which enhance real‑time monitoring, process optimization, and pollutant emission reduction. | Yes |
